# CAR-T cells targeting CLL-1 as an approach to treat acute myeloid leukemia

**DOI:** 10.1186/s13045-017-0553-5

**Published:** 2018-01-10

**Authors:** Jinghua Wang, Siyu Chen, Wei Xiao, Wende Li, Liang Wang, Shuo Yang, Weida Wang, Liping Xu, Shuangye Liao, Wenjian Liu, Yang Wang, Nawei Liu, Jianeng Zhang, Xiaojun Xia, Tiebang Kang, Gong Chen, Xiuyu Cai, Han Yang, Xing Zhang, Yue Lu, Penghui Zhou

**Affiliations:** 1 State Key Laboratory of Oncology in Southern China, Collaborative Innovation Center for Cancer Medicine, Guangzhou, 510060 China; 2 Department of Hematological Oncology, Sun Yat-Sen University Cancer Center, Guangzhou, 510060 China; 3 Guangdong Laboratory Animals Monitoring Institute, Guangdong Key Laboratory of Laboratory Animals, Guangzhou, 510663 China; 4 Department of Hematology, Zhujiang Hospital of Southern Medical University, Guangzhou, 510282 China; 5 Department of Hematology, Sun Yat-Sen Memorial Hospital of Sun Yat-Sen University, Guangzhou, 510120 China; 6 Department of Experimental Research, Sun Yat-Sen University Cancer Center, Guangzhou, 510060 China

**Keywords:** Acute myeloid leukemia, C-type lectin-like molecule-1, Chimeric antigen receptor, Immunotherapy, Leukemia stem cells

## Abstract

**Background:**

Acute myeloid leukemia (AML) is one of the most common types of adult acute leukemia. Standard chemotherapies can induce complete remission in selected patients; however, a majority of patients eventually relapse and succumb to the disease. Thus, the development of novel therapeutics for AML is urgently needed. Human C-type lectin-like molecule-1 (CLL-1) is a type II transmembrane glycoprotein, and its expression is restricted to myeloid cells and the majority of AML blasts. Moreover, CLL-1 is expressed in leukemia stem cells (LSCs), but absent in hematopoietic stem cells (HSCs), which may provide a potential therapeutic target for AML treatment.

**Methods:**

We tested the expression of CLL-1 antigen on peripheral blood cells and bone marrow cells in healthy donor and AML patients. Then, we developed a chimeric antigen receptor (CAR) containing a CLL1-specific single-chain variable fragment, in combination with CD28, 4-1BB costimulatory domains, and CD3-ζ signaling domain. We further investigate the function of CLL-1 CAR-T cells.

**Results:**

The CLL-1 CAR-T cells specifically lysed CLL-1^+^ cell lines as well as primary AML patient samples in vitro. Strong anti-leukemic activity was observed in vivo by using a xenograft model of disseminated AML. Importantly, CLL-1^+^ myeloid progenitor cells and mature myeloid cells were specifically eliminated by CLL-1 CAR-T cells, while normal HSCs were not targeted due to the lack of CLL-1 expression.

**Conclusions:**

CLL-1 CAR-T represents a promising immunotherapy for the treatment of AML.

**Electronic supplementary material:**

The online version of this article (10.1186/s13045-017-0553-5) contains supplementary material, which is available to authorized users.

## Background

Acute myeloid leukemia (AML) is the most common acute leukemia in adults. It is characterized by the accumulation of immature myeloid cells in the bone marrow that results in dysfunction of hematopoiesis [1]. Chemotherapy and hematopoietic stem cell transplantation (HSCT) are the two standard treatments of AML. While improvements have been made in AML treatment in recent decades, the 5-year survival rate remains below 50% due to chemo-resistance or toxicity to these treatments [2, 3]. Most patients eventually succumb to relapsed and/or progressive disease [4], suggesting novel therapeutic strategies are urgently needed for these patients.

Recent advances in immunotherapy have generated substantial excitement for cancer patients. CAR (chimeric antigen receptors)-transduced T cell therapy is one of the new approaches with superior efficacy for the treatment of AML. It combines the specificity of antibody target recognition with the potent effector mechanisms of T cells. CARs are composed of an extracellular antigen-binding domain derived from the single-chain variable fragment (scFv) of the targeting antibody, a transmembrane domain, an intracellular signaling domain of the CD3-ζ, and one or more costimulation domains such as 4-1BB (CD137), CD28, or ICOS (CD278) [5–8]. While the scFv domain mediates target recognition in a major histocompatibility complex (MHC)-independent manner [9–11]. Immune escape through reduction of antigen processing and presentation is not required for CAR-T cells to kill cancer cells. Recently, CAR-T cell therapy has become the most promising approach for leukemia treatment by using lineage specific surface makers of blood cells as targets. For example, CD19-redirected CAR-T cells have been demonstrated with great success in treating CD19^+^ B cell malignancies [12–16]. Its response rate for B cell acute lymphoblastic leukemia (B-ALL) has reached approximately 90%.

In the past decades, a number of tumor antigens such as CD33, CD123, CD44, TIM-3, CD47, and CD32 have been explored as target antigens for AML treatment [17–24]. Although mono-antibody (mAb) therapy targeting these antigens have been shown anti-tumor activity in animal models and clinical trials, the overall therapeutic efficacies remain low. With the success of CD19 CAR-T cell therapy on B-ALL, CAR-T cells targeting these AML associated antigens have been developed and showed higher anti-tumor efficacy compared to mAb therapy. Unfortunately, hematological toxicity was observed due to the expression of these antigens in normal HSCs [25]. Notably, the off-target toxicity of CAR-T therapy may be more severe than traditional antibody-based drugs because of high sensitivity of this therapy and replicative capacity of T cells. Thus, an ideal target for CAR-T therapy against AML should be overexpressed in tumor cells, with minimal or no expression in the HSC compartment and other normal cells.

Human C-type lectin-like molecule-1 (CLL-1, CLEC12A, MICL, KLRL1, or DCAL-2) has been identified as a type II transmembrane glycoprotein that functions as an inhibitory receptor [26–31]. The ligand of this receptor remains to be defined. The expression of CLL-1 is restricted in myeloid lineage cells, as well as in the majority of AML blasts. Leukemic stem cells (LSCs) are regarded as the primary cause of treatment failure and relapse of AML [32]. CLL-1 is selectively present on LSCs in AML but absent in normal HSCs [26, 33, 34], suggesting that CLL-1 is an excellent therapeutic target for AML. Indeed, mAb therapy targeting CLL-1 has been revealed its potential efficacy against AML cells and shown to be effective in reducing AML burden in xenograft model [35, 36].

In this study, we first demonstrated that the CLL-1 antigen is an ideal target of AML for CAR-T therapy displaying restricted expression in myeloid cells and no expression in HSC. We then generated CAR-T cells by using the scFv region of the mAb against CLL-1 coupled to the costimulatory domains of CD28, 4-1BB, and the CD3-ζ chain. The CLL-1 CAR-T cells showed strong therapeutic potential against CLL-1^+^ AML cells in vitro and in vivo. We further observed that HSCs were not targeted because of the lack of CLL-1 expression, thus avoiding toxicity to HSCs.

## Results

### CLL-1 is an ideal target of AML for CAR-T therapy

**Fig. 1:**
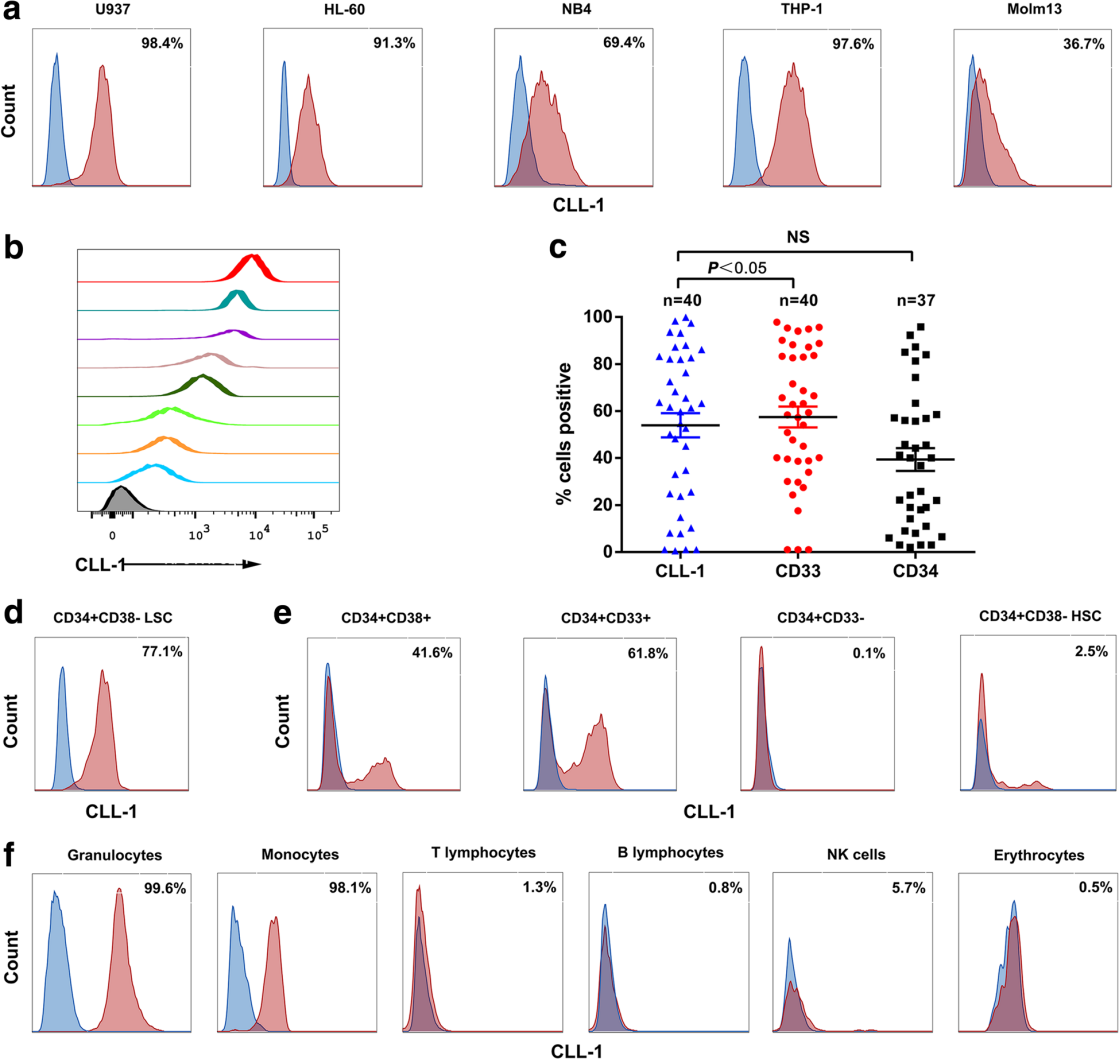
Expression of CLL-1 in AML and normal cells. **a** CLL-1 expression on AML cell lines U937, HL60, NB4, THP-1, and Molm13. The percentages of CLL-1^+^ cells of each sample are indicated. **b** Variant CLL-1 expression levels among primary AML samples. CLL-1 staining from eight representative samples is shown. **c** Distribution of CLL-1^+^, CD33^+^, and CD34^+^ cells in primary AML samples (*n* = 37–40, from a diverse range of AML subtypes; see Additional file 1: Table S1); *NS* not significant. **d** CLL-1 expression on CD34^+^CD38^−^ LSCs within AML bone marrow. **e** CLL-1 expression on CD34^+^ cells within normal bone marrow. **f** CLL-1 expression on normal peripheral blood cells. One representative experiment of three is shown. *HSC* hematopoietic stem cell, *NK* natural killer

CLL-1 has been reported to be expressed in myeloid lineage and AML blasts. In order to determine if CLL-1 is an ideal AML target for CAR-T therapy, we first evaluated CLL-1 surface expression on AML cell lines and primary AML blasts by flow cytometry. As shown in Fig. 1a, a range of CLL-1 expression on AML cell lines U937, HL-60, NB4, THP-1, and Molm13 was detected. We then measured CLL-1 expression on primary AML blasts using 40 fresh or frozen AML specimens including the bone marrow and peripheral blood. The clinical characteristics of the patients are presented in Additional file 1: Table S1. Consistent with previous reports, CLL-1 expression was detected in most AML specimens (77.5%) with different intensities (Fig. 1b), and was detected in AML samples with different FAB subtypes (Additional file 1: Table S1). The representative examples of CLL-1 and CD33 expression on AML blasts are shown in Additional file 2: Figure S1. Since CD33 and CD34 are classic markers for AML, we measured the expression of these two markers in combination with CLL-1 on primary AML samples. We found that CLL-1 is more frequently expressed than CD34 (*p* < 0.05), but no difference with CD33 (Fig. 1c).

We next investigated the expression of CLL-1 in progenitor cells and stem cells from AML patients and healthy donors. CD34^+^ cells from the bone marrow were enriched using magnetic beads and then used for flow cytometric analysis. CD34^+^ cells gated from CD45^dim^ and SSC^low^ cells were subsequently analyzed for the expression of CD33, CD38, and CLL-1 (Additional file 3: Figure S2). We observed that CLL-1 was expressed in most LSCs (CD34^+^CD38^−^) at high levels (Fig. 1d). This suggests that targeting CLL-1 has the potential to eradicate LSCs. Within the bone marrows from three healthy donors, only a minority of the progenitor cells (CD34^+^CD38^+^) expressed CLL-1, while most of the granulocyte and monocyte precursor cells (CD34^+^CD33^+^) expressed CLL-1 at high levels, in contrast CD34^+^CD33^−^ cells showed virtually no expression of CLL-1. Most importantly, HSCs (CD34^+^CD38^−^) was lack of the CLL-1 expression (Fig. 1e). Lymphoid and myeloid subpopulations were segregated on the basis of CD45 staining and side scatter. Granulocytes (CD45^dim^, SSC^high^) as well as monocytes (CD45^dim^, SSC^dim^) expressed CLL-1 antigen in the bone marrow. We further investigated the expression of CLL-1 in peripheral blood cells. Both granulocytes and monocytes expressed CLL-1 antigen. No expression was observed on the T lymphocytes (CD3^+^CD19^−^), B lymphocytes (CD3^−^CD19^+^), and natural killer cells (CD3^−^ CD56^+^), which mirrored the expression profile in the bone marrow (Fig. 1f). Since the M6 subtype of AML is derived from erythrocytes, we further investigated the expression of CLL-1 on erythrocytes (CD235a^+^), but no expression was detected, indicating that CLL-1 might not be a target for AML-M6 (Fig. 1f).

### Generation of CLL-1 CAR-T cells

**Fig. 2:**
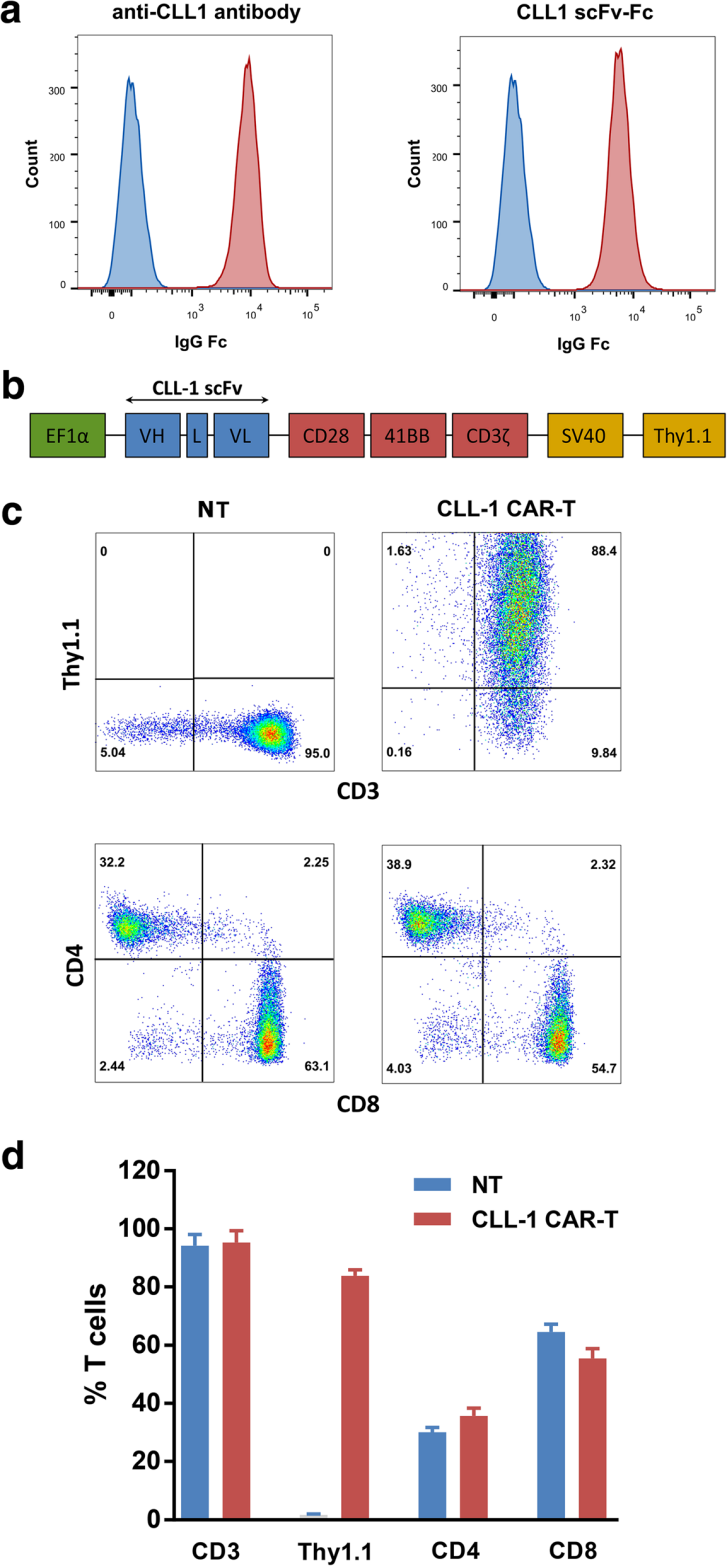
CLL-1 CAR construction and expression in primary human T cells. **a** Cell surface-binding of anti-CLL-1 antibody and CLL-1 scFv-Fc protein to U937 cells, anti-IgG Fc was used as the secondary antibody. **b** Schematic of the CLL-1 CAR vector containing the anti-human CLL-1 scFv linked to CD28, 4-1BB costimulatory domains, and CD3-ζ signaling domain. **c** Representative phenotype of non-transduced T cells (NT) and CAR-transduced T cells derived from a single healthy donor. CAR-modified T cells were stained with anti-CD3 and anti-Thy1.1 after immunomagnetic selection and 1 cycle of expansion. Anti-CD4 and anti-CD8 were analyzed as well. Percentages in each quadrant are indicated. **d** Expression of indicated cell surface markers from three different healthy donor T cell lines following immunomagnetic selection and 1 cycle of expansion. Data represent mean values ± standard deviation (SD). *VH* variable heavy chain, *L* linker, *VL* variable light chain, *scFv* single-chain variable fragment

In order to generate CLL-1 CAR, an anti-CLL1 antibody was generated from C57BL/6 immunized with the CLL-1-Fc fusion protein. The identified antibody showed specific staining on U937 cells (Fig. 2a). The scFv sequence was further fused with Fc to confirm its binding capability. The CLL-1 scFv-Fc showed specific staining on U937 cells (Fig. 2a).

To redirect T cell specificity, we developed lentiviral vectors encoding CLL-1 CAR. The CAR consists of CLL-1 scFv, CD28, 4-1BB costimulatory domains, and CD3-ζ signaling domain. The murine Thy1.1 gene driven by a separated SV40 promoter was inserted downstream of the CAR sequence. It was used as a surface marker and an enrichment target for CAR-expressing T cells (Fig. 2b). OKT3-stimulated peripheral blood CD3^+^ T cells from healthy donors were lenti-transduced, and CAR-expressing T cells were isolated by magnetic selection using a biotinylated-Thy1.1 antibody followed by anti-biotin magnetic beads. After 7 days of culture, the isolated cells were analyzed by flow cytometry for CAR expression and T cell phenotype. The Thy1.1 expression was > 80% in the T cells from three healthy donors. Phenotypic analysis showed that the final CAR-T cells consisted of both CD4^+^ and CD8^+^ T cells, which is comparable to the non-transduced T (NT) cells (Fig. 2c, d).

### CLL-1 CAR-T cells specifically target CLL1-expressing tumor cell lines

**Fig. 3:**
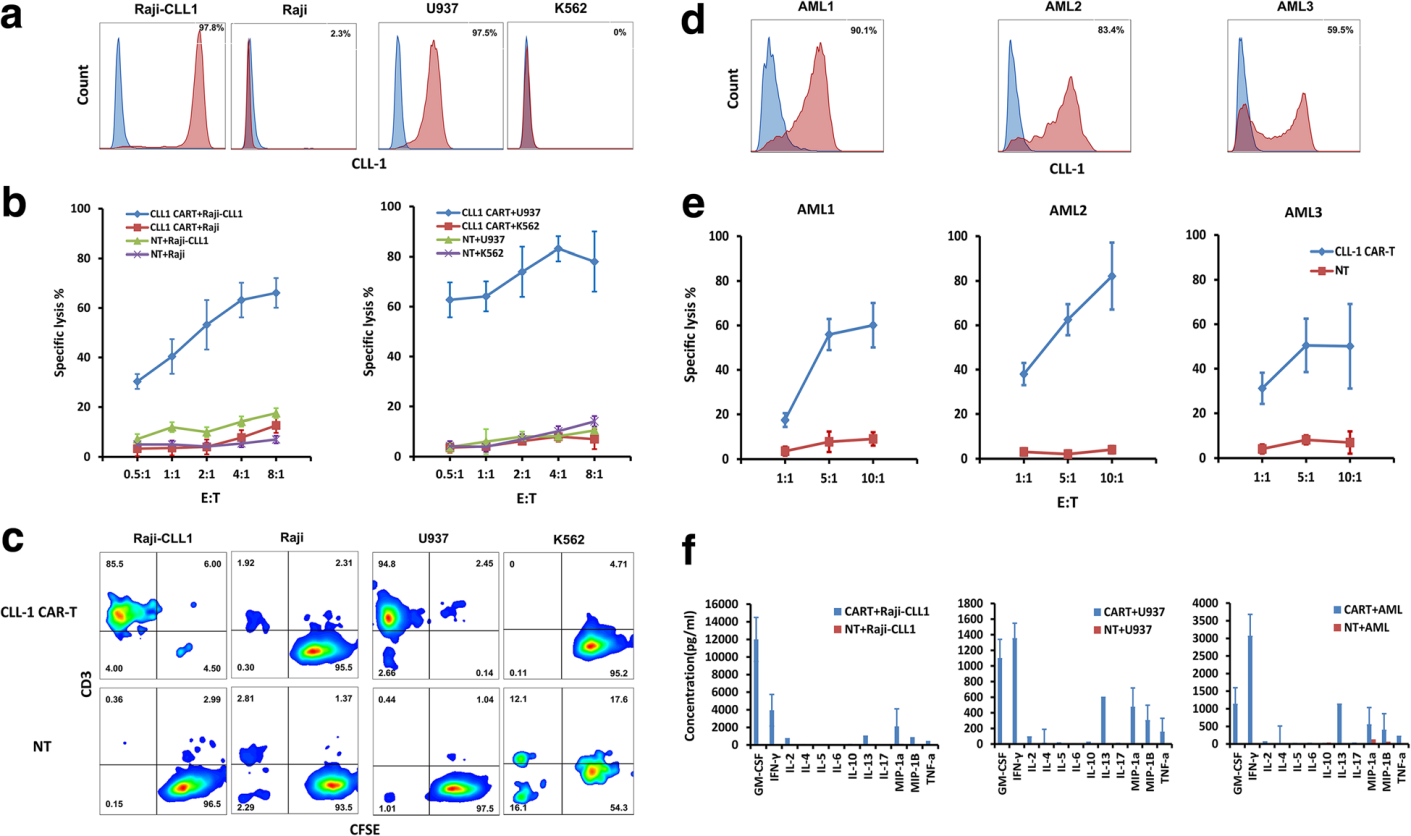
CLL-1 CAR-T cells lyse CLL1-expressing tumor cells. **a** Expression of CLL-1 on the cell line Raji-CLL1, Raji, U937, and K562. **b** CLL-1 CAR-T cells lysed CLL-1^+^ cell lines Raji-CLL1 and U937. CLL-1^−^ cell lines Raji and K562 were used as negative control. NT cells were used to evaluate unspecific lysis. Data represent mean values of triplicate wells ± SD. **c** CLL-1 CAR-T cells underwent proliferation to CLL-1 antigen. CFSE-labeled target cells were co-cultured with CAR-T cells for 4 days in the absence of exogenous cytokines at an E:T ratio of 1:1. Cells were stained using anti-CD3 to distinguish between T cells and CFSE-labeled tumor cells. Percentages in each quadrant are indicated. **d** Expression of CLL-1 on three primary AML samples used in the in vitro killing assay. **e** CLL-1 CAR-T cells displayed cytotoxicity to autologous primary AML cells. CLL-1 CAR was transduced into T cells derived from the patients and cultured with autologous CD34-enriched AML cells for 24 h at the indicated E:T ratios; NT cells were used as negative controls. Data represent mean values of triplicate wells ± SD. **f** Cytokine profiling of CLL-1 CAR-T or NT cells in response to a 24-h co-culture with various target cells. Data represent mean values of triplicate wells ± SD. *GM-CSF* granulocyte macrophage colony-stimulating factor, *IFN-γ* interferon-γ, *IL-13* interleukin-13, *IL-2* interleukin-2, *MIP-1α* macrophage inflammatory protein-1α, *TNF-α* tumor necrosis factor α

To evaluate CLL1-specific reactivity of CLL-1 CAR-T cells, we used two sets of cell lines for the in vitro lysis assay. In the first set, we used the human lymphoma cell line Raji as a negative control as it lacked CLL-1 expression. We then engineered Raji cell line to express CLL-1 cDNA (Raji-CLL1) for use as positive control (Fig. 3a). In the second set, we used the tumor cell lines U937 and HL-60 with endogenous expression of CLL-1 as the positive control, while the chronic myeloid leukemia cell line K562 without CLL-1 expression were used as negative control. The expression of CLL-1 on the cell line Raji-CLL1, Raji, U937, K562, and HL-60 are shown in Fig. 3a and Additional file 4: Figure S3.

CLL-1 CAR-T cells efficiently lysed Raji cells with forced expression of CLL-1 but not the parental Raji cells (Fig. 3b), demonstrating their specific recognition of CLL-1. Similar results were observed when the tumor cell lines U937, HL-60, and K562 were used as target cells. CLL-1 CAR-T cells efficiently lysed CLL-1^+^ U937 and HL-60 cells but not CLL-1^−^ K562 cells (Fig. 3b and Additional file 4: Figure S3). NT cells were used as control to evaluate unspecific lysis. The NT control cells showed weak cytotoxicity against all the four cell lines but no selectivity to CLL-1 expression. In order to evaluate the killing capability of CLL-1 CAR-T cells on CLL-1^+^ cells over time, we performed a long-term cytotoxicity assay. CLL-1 CAR-T cells were incubated with target cells for 96 h in the absence of exogenous cytokines. We observed that CLL-1 CAR-T cells effectively eliminated CLL-1^+^ Raji-CLL1 and U937 cells while sparing the CLL-1^−^ Raji and K562 cells (Fig. 3c). Similar results were observed at effector-to-target (E:T) ratios of 1:1 and 2:1. These data indicated that the engineered CLL-1 CAR-T cells specifically targeted the CLL-1 antigen.

To examine the effector function of CLL-1 CAR-T cells, a panel of cytokines and chemokines were measured during the in vitro cytotoxicity assay. Compared with NT control cells, a broad range of cytokines and chemokines were produced by CLL-1 CAR-T cells when co-cultured with CLL1^+^ target cells. Increased expression of effector cytokines and chemokines such as interferon-γ (IFNγ), tumor necrosis factor-α (TNF-α), granulocyte macrophage colony-stimulating factor (GM-CSF), interleukin-13 (IL-13), and macrophage inflammatory protein α, β (MIP-1α, MIP-1β) was observed (Fig. 3f).

We further evaluate the proliferative potential of CLL-1 CAR-T cells in response to CLL1^+^ cells. Carboxyfluorescein diacetate succinimidyl ester (CFSE) labeled T cells were co-cultured with target cells for 96 h. The decay of CFSE intensity on T cells was used to evaluate the proliferative status by flow cytometry. The results showed that CLL-1 CAR-T cells underwent proliferation when co-cultured with CLL1^+^ U937 cells or primary AML cells, but not with the CLL1^−^ K562 cells. In contrast, the NT controls cells only proliferated when stimulated by OKT3 (Additional file 5: Figure S4). These data suggested that the engineered CLL-1 CAR-T cells are able to execute an effective immune attack against CLL-1^+^ tumor cells.

### CLL-1 CAR-T cells specifically target primary AML blasts

Next, we used T cells and AML cells from 3 CLL-1^+^ AML patients to determine if CLL-1 CAR-T cells could lyse autologous primary AML cells in vitro. A representative example of the CLL-1 antigen expression is shown in Fig. 3d (AML 1–3). CD3^+^ T lymphocytes isolated from the peripheral blood of the three patients were stimulated with anti-CD3 and anti-CD28 antibodies and then were lentivirally transduced to express CLL-1 CAR. The transduction efficiency among the three patients was varied from 30 to 70% (Additional file 6: Figure S5). The cytolytic potential of patient-derived CLL-1 CAR-T cells against autologous AML cells (CD34 enriched) was evaluated after a 24-h co-culture. CLL-1 CAR-T cells derived from patients 1 and 2 efficiently lysed autologous blasts, while patient 3 displayed lower level of lysis probably due to the lower and heterogeneous expression of CLL-1 on his/her AML blasts (Fig. 3e).

Consistent with the previous in vitro cytotoxicity assay using cell lines as targets, increased expression of effector cytokines and chemokines were observed when patient-derived CLL-1 CAR-T cells and CLL-1^+^ autologous AML cells were co-cultured (Fig. 3f).

### CLL-1 CAR-T cells eliminate human AML in xenograft models

**Fig. 4:**
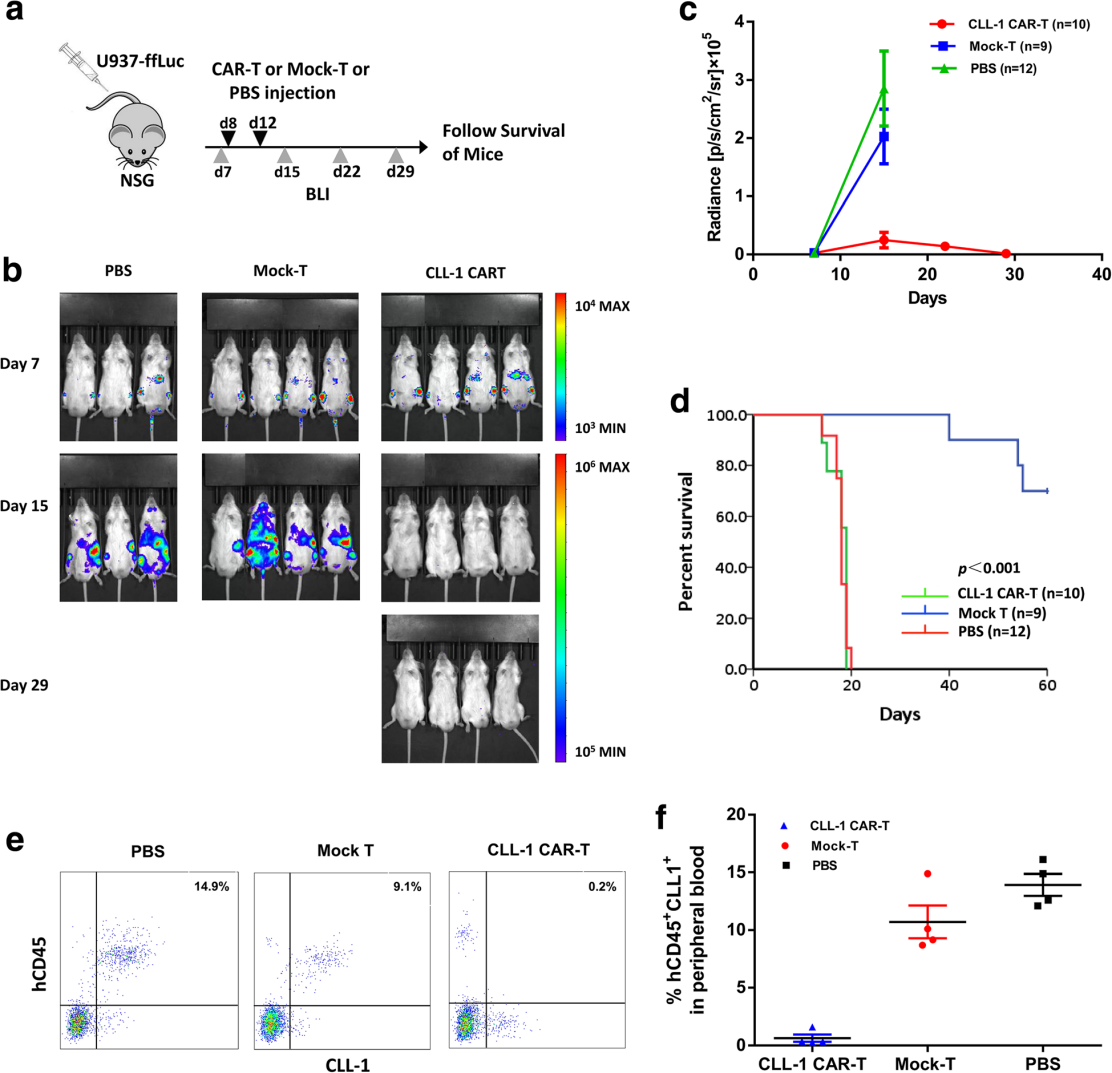
CLL-1 CAR-T cells eliminate human AML in xenograft models. **a** Schematic of the U937 xenograft model. NSG mice were injected via tail vein with 1 × 10^6^ U937-firefly luciferase (U937-ffLuc) on day 1. Bioluminescent imaging (BLI) was performed on day 7 to quantify engraftment and for randomization of treatment groups. CLL-1 CAR-T cells (1–1.5 × 10^6^), Mock T cells (1–1.5 × 10^6^), or PBS were injected iv on day 8 and day 12, and mice were followed with serial BLI. Quantification of BLI radiance was used as a surrogate measurement of AML burden. **b** BLI prior to T cell treatment (day 7), on day 15, and on day 29 following U937-ffLuc transplantation. **c** Bioluminescent signal for each treatment group over time. Data represent mean values of each group ± SD. Results represent pooled data from three separate experiments. **d** Kaplan-Meier analysis of survival. Log-rank (Mantel-Cox) tests were used to perform statistical analyses of survival between groups. Data were summarized from three independent experiments. **e** Representative flow cytometric analysis of peripheral blood 18 days after leukemia transplant. Percentage of human CD45^+^ CLL-1^+^ U937 cells is indicated. **f** Summary of leukemic cell engraftment in mouse peripheral blood 18 days after leukemia transplant. The percentage of human CD45^+^ CLL-1^+^ U937 cells is indicated. Each symbol indicates one mouse

After confirming the specificity and reactivity of CLL-1 CAR-T cells against human AML in vitro, we further assessed their anti-leukemic activity in vivo. We first developed a xenogeneic model of systemic AML using the U937 leukemic cell line engineered to express the firefly luciferase reporter gene (U937-ffLuc). NOD/SCID IL-2RγC^null^ (NSG) mice were inoculated with 1 × 10^6^ U937-ffLuc cells intravenously on day 1. On days 8 and 12 after tumor inoculation, mice were treated with intravenous injection of 1–1.5 × 10^6^ CLL-1 CAR-T or mock T cells or phosphate-buffered saline (PBS) (Fig. 4a). Tumor progression was monitored by bioluminescent imaging (BLI) once a week beginning on day 15. Control mice treated with mock T cells or PBS showed a rapid progression of leukemia. Most of them died around day 20. In contrast, mice treated with CLL-1 CAR-T cells displayed a significant decrease in systemic leukemic burden as evidenced by BLI (Fig. 4b). Signal intensity was plotted over time in Fig. 4c. This resulted in a significant survival advantage for mice treated with CLL-1 CAR-T cells compared with both mock T-treated (*p* < 0.001) and PBS-treated (*p* < 0.001) groups (Fig. 4d). We also investigated the CD45^+^CLL-1^+^ leukemia cells in peripheral blood at day 18 after tumor inoculation. Consistent with a mounting antitumor response, mice treated with CLL-1 CAR-T cells exhibited significantly decreased tumor cells compared to mock T or PBS-treated mice (Fig. 4e, f), and the hCD45^+^ CLL1^−^ population in peripheral blood of CAR-T-treated mice was all human T cells (Additional file 7: Figure S6).

### CLL-1 CAR-T cells do not target hematopoietic stem cells

It has been reported that recognition of the low levels of target antigen on normal tissues by CAR molecules might cause severe toxicity. In addition, many surface markers exploited for CAR therapy of AML including CD123 and CD33 are shared between AML blasts and normal HSCs. CAR-T cells targeting these HSC-shared antigens will impair normal hematopoietic function, thus resulting in severe hematological toxicity.

**Fig. 5:**
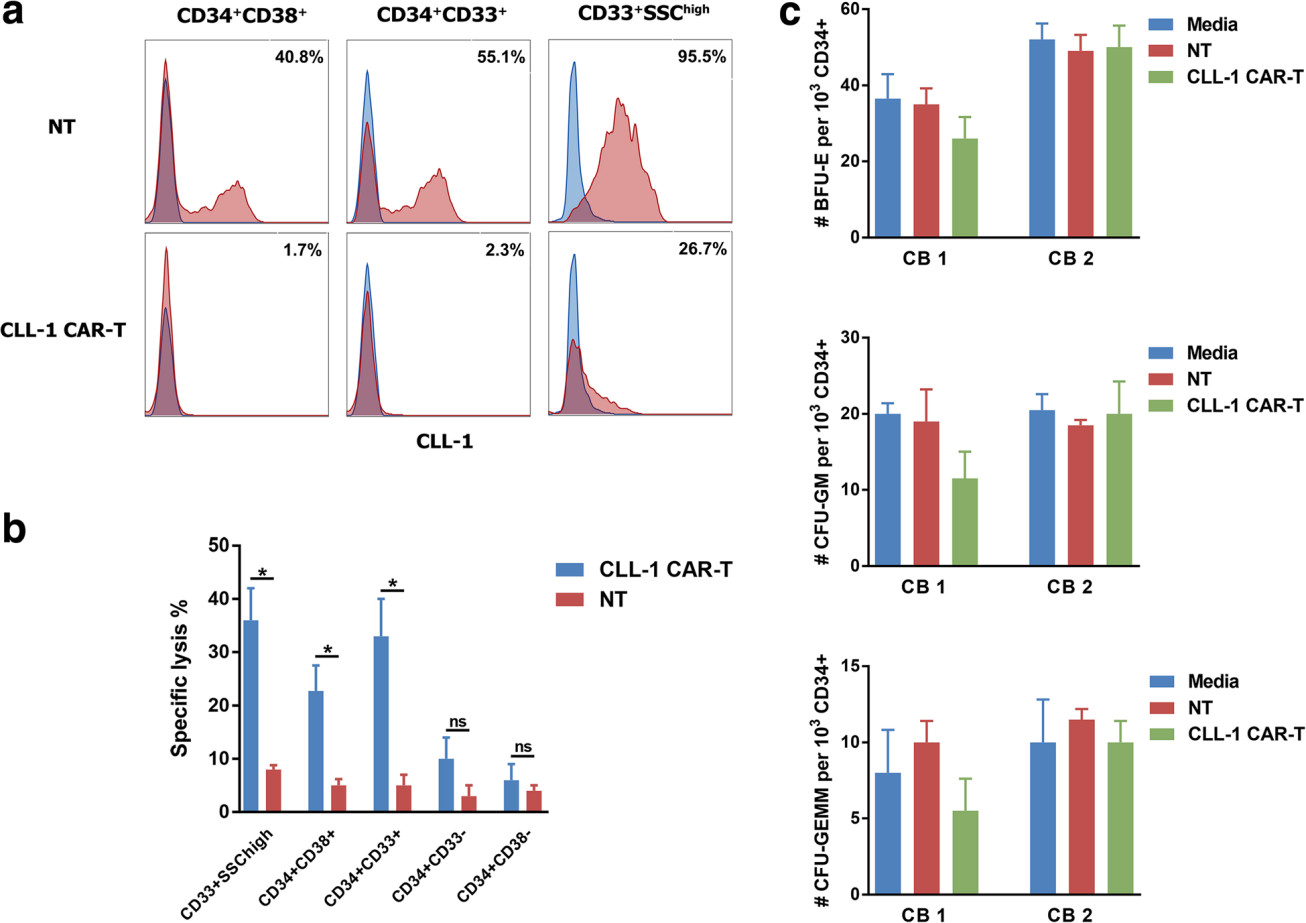
CLL-1 CAR-T cells do not target hematopoietic stem cells. **a** Expression of CLL-1 in mature myeloid cells after co-cultured with CLL-1 CAR-T cells. CD34-enriched normal hematopoietic cells and granulocytes were stained with CLL-1 after 24-h co-cultured with CAR-T cells or NT cells. **b** Summary of the in vitro killing assay for normal mature myeloid cells and HSCs. Normal mature myeloid cells (CD33^+^SSC^high^) and myeloid progenitor cells (CD34^+^CD38^+^/CD34^+^CD33^+^) were lysed by CLL-1 CAR-T cells. Normal HSCs (CD34^+^CD38^−^) were not targeted by CLL-1 CAR-T cells. Data represent mean values of triplicate wells ± SD. **c** CD34^+^ CB cells (*n* = 2) were CD34-immunomagnetically selected and co-cultured with either non-transduced- or CLL-1-specific pair-matched T cells from healthy donors or media alone for 4 h at an E:T of 10:1. The cells were then plated in semisolid methylcellulose-based growth medium for 14 days and counted for the presence of BFU-E, CFU-GM, and CFU-GEMM colonies. Data represent mean values ± SD for two different CB samples. *p < 0.05, *NS* not significant

In order to evaluate the possible side toxicity of CLL-1 CAR-T cells, we assessed the killing effect of CLL-1 CAR-T cells on autologous normal hematopoietic cells and mature myeloid cells. CLL-1 CAR-T cells generated from healthy donors were co-cultured with autologous CD34-enriched bone marrow cells or peripheral blood leukocytes. We found that both CLL1^+^ progenitor cells in the bone marrow and mature granulocytes in peripheral blood were effectively eliminated after a 24-h co-culture at an E:T ratio of 1:1, as evidenced by the lack of CLL-1 expression on residual viable cells (Fig. 5a). The killing effect of CLL-1 CAR-T cells on autologous normal hematopoietic cells and mature myeloid cells are indicated in Fig. 5b. Consistent with the expression level of CLL-1, CLL-1 CAR-T cells eliminated mature granulocytes and CD34^+^CD38^+^ or CD34^+^CD33^+^ myeloid progenitor cells in variable degrees while sparing the CD34^+^CD33^−^ cells. More importantly, CD34^+^CD38^−^ HSCs were not targeted by CLL-1 CAR-T cells due to the lack of CLL-1 expression.

We further investigated the effect of our engineered T cells on the colony-forming ability of CD34-enriched normal cord blood (CB) samples. After 4-h co-culture of CD34^+^ HSCs and CAR-T cells, each well was diluted in methylcellulose and cultured for 14 days. Colonies were counted for burst-forming unit erythroid (BFU-E), colony-forming unit granulocyte-macrophage (CFU-GM), and colony-forming unit granulocyte-erythrocyte-macrophage-megakaryocyte (CFU-GEMM). Interestingly, there were no significant differences in the number of lineage-specific colonies compared to media alone (untreated) or non-transduced T cell-treated controls (Fig. 5c). These findings suggest that the engineered CLL-1 CAR-T cells can be safely pursued as a therapy for AML without the potential toxicity associated with HSC targeting.

## Discussion

CAR-T therapy has achieved great success in a variety of malignant diseases, particularly for hematological cancer in which lineage markers of restricted expression are used as target antigens. For example, CAR-T therapy targeting the B cell lineage marker CD19 has shown striking efficacy in treating CD19^+^ B cell leukemia. As a myeloid malignant disease, AML express multiple myeloid-specific lineage markers such as CD13, CD14, CD117, CD33, and CD123. Subsequently, antibodies targeting some of these antigens have been developed to treat AML [17, 37]. However, limited therapeutic efficacy was observed as well as hematological toxicity, owing to target expression by normal HSCs. CAR-T cells targeting these same antigens provided a more effective approach to eliminate cancer cells expressing these proteins; however, they do not get around the toxicity issue for targets expressed on normal cells. Indeed, CAR-T therapy targeting tumor associated antigens like HER2 showed severe side effects on normal cells [38, 39]. As a result, selecting tumor antigens for CAR-T therapy requires even greater cancer cell specificity that given the T cells greater capacity to recognize cells expressing low amounts of target antigen. In the current study, we confirmed that CLL-1 is selectively expressed on AML blasts but not on normal HSCs and lymphoid lineages, which ensured its safety as an antigen for CAR-T therapy. AML is generally recognized as a stem cell disease. LSCs in AML displayed intrinsic resistance to chemotherapy which results in refractoriness and relapse ultimately [40]. Interestingly, we found CLL-1 is expressed in LSCs as well, further supporting its high potential for AML treatment. On the whole, targeting CLL-1 will not only eradicate the AML cells but also the LSCs, while sparing the normal HSCs. To this end, we generated a CLL-1 antibody and subsequently developed it into a CAR that redirected T cell specificity to CLL-1. The capabilities of CAR-T cells with respect to antigen-specific killing, proliferation, and cytokine secretion were comprehensively evaluated using in vitro killing assays. As expected, the engineered CLL-1 CAR-T cells displayed strict CLL-1 specificity both on AML cell lines and on primary patient samples. Strong anti-leukemic activity was shown in an in vivo xenograft model as well. More importantly, HSC toxicity was not observed due to its lack of CLL-1 expression. We thus demonstrated that the CLL-1 CAR-T cells are a safe therapy with high potential for AML treatment.

In a cohort of 40 AML patients, we found that CLL-1 expression was detected in most AML specimens (77.5%) with different FAB subtypes, supporting its wide application for AML treatment. Nevertheless, variable expression levels of CLL-1 were observed in different patients. In particular, we found no expression of CLL-1 on erythrocyte, indicating that CLL-1 CAR-T cells may not be appropriate for patient with M6 developed from erythrocyte, unless accompanied by abnormal myeloid hematopoietic cell proliferation. Due to the low incidence and difficulty to diagnose morphologically, specimens of AML-M7 were not included in this study. Future study of the CLL-1 expression on AML-M7 is necessary to clarify if CLL-1 CAR-T cells are appropriate for this subtype of AML. In addition, slightly reduced cytotoxicity on the primary AML specimen with low CLL-1 expression was observed in the in vitro killing assay. A possible explanation for this is that CLL-1 expression levels might affect the targeting efficacy of CAR-T cells, though we cannot rule out the variant T cell functions from different patients. Development of CARs with high affinity might be an alternative approach to resolve this potential drawback.

Given the expression of CLL-1 in the normal myeloid lineage, it is very likely that CLL-1 CAR-T cells will eliminate mature myeloid cells such as granulocyte, macrophage, and monocytes, thus interfering with the functions undertaken by myeloid lineage. We found that normal HSCs and lymphoid lineages do not express CLL-1 and verified that CLL-1 CAR-T cells did not eliminate these cells. Therefore, the adaptive immunity mainly carried out by lymphoid lineages will preserve during the treatment. In order to recover hematopoiesis, however, it is necessary to remove CLL-1 CAR-T cells after treatment. Alternative approaches such as incorporation of suicide genes or molecular switches into CAR vectors are able to resolve this disadvantage [41, 42].

Consistent with our study, a recent publication from Tashiro et al. also reported that CLL-1 is a promising target for CAR-T cells to treat AML [43]. We measured CLL-1 expression with other two AML classic markers CD33 and CD34 and found that CLL-1 is more frequently expressed than CD34. In our study, the expression of CLL-1 was detected in 77.5% of the AML specimens with different intensities, while Tashiro et al. observed that 85–92% of their samples were CLL-1-positive. This might be due to the difference of ethnic groups and/or the sizes of samples. We analyzed 40 Chinese patients while they only used 19 French-American-British patients. We both observed that CLL-1 was expressed in most LSCs at high levels but absent in HSCs. Interestingly, the CAR-T cells generated in our study seems be more effective. Tashiro’s report showed an incremental improvement in survival analyze using 0.6 × 10^6^ CLL-1 CAR-T cells to treat irradiated mice injected with 5 × 10^4^ HL-60 AML cells. In our study, 1–1.5 × 10^6^ CLL-1 CAR-T cells were able to treat the disease in mice injected with 1 × 10^6^ U937 AML cells. It might be resulted from the differences in the affinity of scFvs, vector designs, or distinct T cell transduction and expansion strategies.

Along with the rapid progress of CAR-T therapy, resistance to treatment has become a major barrier for its clinical application. For example, the Novartis CTL109 trial has shown a remarkable response rate of 93%. However, its 1 year complete response rate decreased to around 55%, suggesting that almost half of the patients developed resistance in a year. Target antigen loss accounted for most of the resistance in CAR-T therapy. In order to overcome that, using CAR targeting multiple antigens is a well-accepted strategy in the field [44]. In consideration of the variant expression level of CLL-1 in AML patients, a combination with CAR targeting other antigens in AML will definitely enhance the therapeutic effect. Future testing of the co-expression of CLL-1 with other AML antigens such as CD123 and CD117 could provide more options of combinational therapy.

The combination of CLL-1 CAR-T cells with standard chemotherapy is another potential approach to enhance its therapeutic effect [45]. High rate of tumor burden not only increases the difficulty of treatment but also prone to induce side effects like cytokine release syndrome and tumor lysis syndrome due to over-reacted immune attack. Moreover, pretreatment with chemotherapy is able to increase the graft rate of CAR-T cells into host [46]. Further introduction of chemo-resistant genes into CLL-1 CAR vector would enable the CAR-T cells to work with chemotherapy side by side.

Overall, we demonstrated that CLL-1 is an ideal antigen of CAR-T therapy for AML. Our CLL-1 CAR-T cells displayed strong anti-leukemic effects both in vitro and in vivo, while having safe profile on normal HSCs. The CLL-1 CAR-T therapy may be a promising approach to treat AML.

## Conclusions

CLL-1 CAR-T cells displayed strong anti-leukemic effects both in vitro and in vivo, while having safe profile on normal HSCs. The CLL-1 CAR-T therapy may be a promising approach to treat AML.

## Methods

### Antibody generation

To prepare the CLL-1-Fc fusion protein, the extracellular domain of human CLL-1 fused with mouse Fc was synthesized (Synbio, Suzhou, China) and cloned into the lenti-vector pELNS with the EF1α promoter. The CLL-1-Fc fusion protein was purified from the culture supernatant of the CLL-1-Fc-transduced CHO cells using protein G-Sepharose column (GE Healthcare Bio-Sciences, Pittsburgh, Pennsylvania, USA) and dialyzed in PBS.

A mouse mAb against CLL-1 was generated by immunizing a BALB/c mouse (Vital River, Beijing, China) with the CLL-1-Fc fusion protein. The supernatants of hybridomas were screened using FACS to test the binding to U937 cells. The hybridoma secreting CLL-1-binding mAb was selected for further experiments.

### T cell transduction

The DNA sequence of the scFv derived from the CLL-1 antibody was synthesized and cloned into the lenti-vector pELNS. CD28, 4-1BB, and CD3-ζ signaling domains were then constructed into to generate the CLL-1 CAR. Thy1.1 was constructed into as a reporting marker. The lentiviral supernatants were produced by transfecting 293 T cells and concentrated by ultra-centrifuging. The concentrated CLL-1 CAR lenti-virus was immediately stored at − 80 °C for further use.

Healthy donor-derived peripheral blood mononuclear cells (PBMCs) or CD3^+^ enriched T cells (Miltenyi) were expanded in vitro using anti-CD3/CD28 mAbs (Ebioscience) and recombinant human interleukin-2 (IL-2) at 20 ng/ml (Peprotech) for 48 h. The activated T cells were then transduced with lentiviral supernatants on day 3 in plates pre-coated with retronectin (Takara). After transduction, T cells were cultured with IL-2, IL-7, and IL-15. CAR expression on T cells was measured 72 h later, and the cells were isolated by immunomagnetic selection using a biotinylated-Thy1.1 antibody followed by a secondary stain with anti-biotin magnetic beads (Biolegend).

### Cell lines and primary AML samples

The following AML cell lines were purchased from the ATCC: U937, HL-60, NB4, THP-1, and Molm13. K562 cell line (ATCC), negative for CLL-1, was used as a negative control. The Raji cell line (ATCC) was transduced with lentiviral vectors encoding human CLL-1 DNA to generate Raji-CLL1 cells. All the cells were cultured with RPMI 1640 media (Gibco) containing 10% fetal calf serum, penicillin, and streptomycin. The 293 T (ATCC) cells for lentiviral packaging was cultured in DMEM media (Gibco). In the bioluminescent xenograft models, U937 cells were transduced with a lentiviral firefly luciferase construct. Primary human AML specimens were acquired from the SunYat-sen University Cancer Center. This study design was approved by the SunYat-sen University Cancer Center Research Ethics Board. Written informed consent for publication of their clinical details was obtained from the patient/relative of the patient.

### Flow cytometry

All the antibodies were purchased from Biolegend. Cells were washed once in 100 μL of PBS containing 2% fetal bovine serum and labeled on ice after blockade of Fc receptors. Normal bone marrows were treated with erythrocyte lysate. Primary AML cells were isolated through density gradient centrifugal assay and then remaining red blood cells were lysed. A sample was considered positive for CLL-1, CD33, or CD34 if > 20% of the sample cells expressed the antigen (compared to the control sample). For the expression analysis of CLL-1 on CD34^+^ progenitor/stem cell subsets, CD45-PerCP, CD34-FITC, CD38-PE/cy7, CD33-PE, and CLL1-APC mAbs were stained to identify different subpopulations. The transduction rate of CLL-1 CAR into T cells was detected by staining with a mouse Thy1.1 antibody. Flow cytometry were performed on a BD Fortesa flow cytometer, and results were analyzed using the software FlowJo7.6.5.

### In vitro cytotoxicity assay

CFSE-labeled target cells were incubated at the indicated E:T ratios with CLL-1 CAR-T cells plated in triplicate wells for 24 h in RPMI 1640 media. Total cells were harvested and then labeled with 7-AAD for flow cytometry analysis.

### T cell proliferation

T cells were washed and resuspended in PBS at a concentration of 1 × 10^6^/ml and then labeled with 1 μM CFSE (Life Technologies) in PBS for 15 min at 37 °C. The CFSE-labeled T cells were washed and incubated with target cells in the absence of exogenous IL-2 for 96 h. CFSE dilution was quantified by flow cytometry.

### Cytokine secretion

Effector cells and target cells were cultured at an E:T ratio of 1:1 in RPMI 1640 media for 24 h. Supernatant of culture was analyzed by 30-plex Luminex array according to the manufacturer’s instructions (Milliplex).

### Xenograft animal model

All the animal experiments were approved by the Institutional Animal Care and Use Committee (IACUC) of Guangdong Laboratory Animal Monitoring Institute, and the animal facility were accredited by the American Association for Accreditation of Laboratory Animal Care (AAALAC). Schematic of the used xenograft models are delineated in the relevant figures in “Results.” Leukemia engraftment was defined as > 1% human CD45^+^ cells in the peripheral blood by flow cytometry. Mice were sacrificed when moribund or upon the development of hind-limb paralysis. For in vivo imaging of ffLuc cells, mice were injected intraperitoneally with D-luciferin (150 mg/kg) and imaged under isoflurane anesthesia. Mice were analyzed using the Xenogen-IVIS imaging system and quantified with the Living Image software (PerkinElmer).

### Colony-forming cell assay

CD34^+^ cells from CB mononuclear cells were isolated from healthy donors using immunomagnetic column separation (Miltenyi). A total of 1 × 10^3^ CD34^+^ CB cells were co-cultured with non-transduced T or CLL-1 CAR-T cells or media alone for 4 h at an E:T of 10:1, respectively. At the end of the 4-h co-culture, the entire cell mixture was transferred to a semisolid methylcellulose-based growth medium and plated in duplicate. After 14 days, BFU-E, CFU-GM, and CFU-GEMM colonies were enumerated.

### Statistical analysis

The data are presented as mean ± standard deviation (SD). The Student *t* test was used to determine the statistical significance of differences between samples. Analyses were performed using SPSS version 19 statistical software, *p* < 0.05 was considered significant.

## Additional files

Additional file 1: Table S1. Patient characteristics and CLL-1 expression of primary AML patient sample. F, female; M, male; BM, bone marrow; PB, peripheral blood. (DOCX 25 kb)

Additional file 2: Figure S1. Co-expression CLL-1 and CD33 in primary AML samples. (A) Initially, cells were gated based on forward and side scatter properties. Subsequently, AML blasts were selected based on low side scatter versus CD45^dim^ expression. (B) CLL-1 and CD33 expression on four representative gated AML blast cell populations are depicted. Percentages in each quadrant are indicated. (TIFF 1776 kb)

Additional file 3: Figure S2. The gating strategy of CD34^+^ AML blasts. Cells were initially gated based on forward and side scatter properties. Subsequently, AML blasts were selected based on low side scatter versus CD45^dim^ expression. Then, CD34^+^ cells were gated. Finally, CD38^+^/CD38^−^ cells were gated and used for CLL-1 expression analysis. (TIFF 1656 kb)

Additional file 4: Figure S3. CLL-1 CAR-T cells lyse CLL1-expressing AML cells. (A) Expression of CLL-1 on the cell lines HL-60 and K562. (B) CLL-1 CAR-T cells lysed CLL-1^+^ cell line HL-60. CLL-1^−^ cell line K562 was used as negative control. NT cells were used to evaluate unspecific lysis. Data represent mean values of triplicate wells ± SD. (TIFF 481 kb)

Additional file 5: Figure S4. Proliferation of CLL-1 CAR-T cells in response to CLL-1^+^ cells. Pair-matched CFSE-labeled CLL-1 CAR-T cells or NT cells were co-cultured with the indicated stimulator cell lines for 96 h at an E:T of 1:1. CFSE dilution was analyzed by flow cytometry. Unstimulated T cells (gray histograms) were used as baseline T cell proliferation controls. (TIFF 866 kb)

Additional file 6: Figure S5. CLL-1 CAR expression in T cells derived from AML patients. T cells from three AML patients were transduced with CLL-1 CAR. Shown are CLL-1 CAR-T and NT cells from the three AML patients 14 days post transduction. Percentages in each quadrant are indicated. (TIFF 557 kb)

Additional file 7: Figure S6. Representative flow cytometric analysis of peripheral blood of CAR-T-treated mice. Eighteen days after leukemia transplant, hCD45^+^ CLL1^−^ population in peripheral blood of CAR-T-treated mice was almost human T cells (hCD45^+^ CD3^+^). (TIFF 4614 kb)

## Abbreviations

AML: Acute myeloid leukemia
B-ALL: B Cell acute lymphoblastic leukemia
BFU-E: Burst-forming unit erythroid
BLI: Bioluminescent imaging
CAR: Chimeric antigen receptor
CB: Cord blood
CFSE: Carboxyfluorescein diacetate succinimidyl ester
CFU: Colony-forming unit
CLL-1: C-type lectin-like molecule-1
GM-CSF: Granulocyte macrophage colony-stimulating factor
HSCT: Hematopoietic stem cell transplantation
IFNγ: Interferon-γ
IL-13: Interleukin-13
LSCs: Leukemic stem cells
mAb: Mono-antibody
MHC: Major histocompatibility complex
MIP-1α: Macrophage inflammatory protein α
MIP-1β: Macrophage inflammatory protein β
NSG: NOD/SCID IL-2RγC^null^
NT: Non-transduced T
PBMCs: Peripheral blood mononuclear cells
PBS: Phosphate-buffered saline
scFv: Single-chain variable fragment
SD: Standard deviation
TNF-α: Tumor necrosis factor-α
