# Supplementary material for: CAR-T cells targeting CLL-1 as an approach to treat acute myeloid leukemia
Source: J Hematol Oncol. 2018 Jan 10;11:7. doi: 10.1186/s13045-017-0553-5 (PMC5761206; doi:10.1186/s13045-017-0553-5)
Supplement: Supplementary file 1 — Patient characteristics and CLL-1 expression of primary AML patient sample. F, female; M, male; BM, bone marrow; PB, peripheral blood. (DOCX 25 kb) [file 13045_2017_553_MOESM1_ESM.docx]

| No. | Sample source | Sex | Age | Status | Subtype | WBC(×10^9^) | Blast % | Cytogenetics_Abnormality | All Flow Results List | CLL-1% positive | CD33% positive | CD34% positive |
| --- | --- | --- | --- | --- | --- | --- | --- | --- | --- | --- | --- | --- |
| 1 | BM | male | 57 | new diagnosed | M3 | 101.71 | 93.7 | PML/RARA+ | MPO+CD117+CD33+CD13+CD64+CD38+CD56+CD2+CD34-HLA-DR-CD15-CD11b-CD16-CD10-CD20-CD14-CD11e-CD4-CD8-CD3-CD19-CD5-CD7-CD22-TdT-eCD3- | 98.3 | 94 | 11 |
| 2 | BM | female | 31 | new diagnosed | M2 | 6.28 | 29.8 |  | CD33+CD13+CD117+HLA-DR+MPO+CD64+CD56+CD11b+CD16+CD15-CD16-CD10-CD20-CD14-CD11b-CD11e-CD4-CD8-CD3-CD19-CD2-CD5-CD7-CD22- | 34.9 | 40.1 |  |
| 3 | BM | male | 28 | new diagnosed | M3 | 2.24 | 85.5 | PML/RARA+ | CD117+CD33+CD13+CD64+MPO+CD9+HLA-DR+CD15-CD34-CD36-CD123-CD16-CD11b-CD10-CD20-CD14-CD11e-CD4-CD8-CD3-CD19-CD56-CD7-CD22-CD2-CD5-TdT-eCD3- | 25.6 | 40.1 | 9 |
| 4 | BM | female | 27 | new diagnosed | M2 | 63.43 | 41.2 | NPM1+,FLT3-ITD Wildtype | CD33+CD13+CD15dim+CD34+CD117+HLA-DR+CD7+MPO+CD64+CD56+CD15+CD16+CD11b-CD16-CD10-CD20-CD14-CD11e-CD4-CD8-CD3-CD19-CD2-CD5-TdT-eCD3-CD117-CD34-HLA-DR-CD20-CD11c-CD22-CD7- | 82 | 71.6 | 41.2 |
| 5 | BM | male | 24 | new diagnosed | M3 | 15.22 | 81.9 | PML/RARA+ | CD33+CD13+CD615+MPO+CD9+CD117+CD11b+CD64+CD11c+CD34+CD2+HLA-DR-CD16-CD10-CD20-CD14-CD4-CD8-CD3-CD19-CD56-CD5-CD7-CD22-TdT-eCD3-CD36- | 86.2 | 83.4 | 40 |
| 6 | BM | female | 30 | new diagnosed | M5 | 373.9 | 96 | FLT3-ITD [mutation](javascript:void(0);)，NPM1 Wildtype | CD64+CD11e+CD33+CD117+HLA-DR+CD34+CD13+CD15+CD11b+MPO+CD14-CD16-CD10-CD20-CD4-CD8-CD3-CD19-CD56-CD22-CD7-CD2-CD5-TdT-eCD3- | 93.2 | 88.9 | 57.1 |
| 7 | BM | male | 61 | new diagnosed | M5 | 42.92 | 56 |  | CD117+CD34+HLA-DR+CD13+MPO+CD15+CD33+CD9+CD14+CD64+ CD11e+CD11b+CD300e+CD36+CD11b-CD14-CD11e-CD64-CD16-CD10-CD20-CD3-CD19-CD56-CD4-CD8-CD22-CD5-CD7-CD2-eCD3-TdT-CD36- cD123-CD300e-CD117-CD34- | 82.7 | 45 | 56 |
| 8 | BM | female | 48 | new diagnosed | M2 | 86.97 | 73 |  | CD34+CD117+CD33+CD13+CD64dim+CD9+CD123+CD38+CD15dim+CD11b+CD16-HLA-DR-CD10-CD20-CD11c-CD14-CD4-CD8-CD56-CD19-CD3-CD7-CD2-CD22-CD5-MPO-TdT-eCD3-CD61-CD42b-CD36-CD41- | 52.7 | 90.2 | 63.4 |
| 9 | BM | female | 38 | new diagnosed | M5 | 345.6 | 91.5 |  | CD33+CD13+CD7+CD34+CD117+HLA-DR+CD56+MPO+CD64+CD38+CD11b+CD15- CD16-CD10-CD20-CD14-CD11e-CD4-CD8-CD3-CD19-CD2-CD5-CD22-TdT-eCD3- | 54.7 | 57.3 |  |
| 10 | BM | male | 60 | new diagnosed | M5 | 205.76 | 81.4 |  | CD34+CD117+CD33+CD13+MPO-CD15-HLA-DR-CD38-CD16-CD10-CD20-CD11b-CD14-CD64-CD11e-CD4-CD8-CD56-CD19-CD3-CD2-CD5-CD7-CD22-TdT-eCD3-CD123- | 88 | 94.9 | 81.4 |
| 11 | BM | female | 53 | new diagnosed | M5 | 4.79 | 22.3 | 7q- | CD34+HLA-DR+CD7+CD117+CD64-CD14-CD19- | 1 | 1 | 22 |
| 12 | BM | female | 32 | relapse | M5 | 1.82 | 15 | AML1-ETO+ | CD34+CD117+HLA-DR+CD15+CD56+CD11b-CD19- | 50 | 29.8 |  |
| 13 | BM | male | 54 | new diagnosed | M5 | 11.95 | 22.2 |  | CD34+CD117+CD38+CD13+HLA-DR+CD11c+TdT+CD15+CD33+CD11b+CD64+CD14+CD11e+CD15-CD16-CD10-CD20-CD14-CD64-CD4-CD8-CD56-CD3-CD19-CD7-CD22-CD2-CD5-CD123-MPO- | 45 | 39.5 | 19 |
| 14 | BM | male | 20 | new diagnosed | M5 | 1.93 | 9.5 |  | HLA-DR+CD117+CD64+CD14+CD64brl+CD434-CD7-CD19-CD34-CD117- | 99.9 | 38.8 | 19 |
| 15 | BM | female | 54 | relapse | M2 | 2.59 | 25.5 |  | CD15dim+CD117+CD33+CD13dim+MPO+CD34-HLA-DR-CD16-CD10-CD20-CD11b-CD14-CD64-CD11e-CD38-CD4-CD8-CD56-CD3-CD19-CD2-CD5-CD7-CD22-TdT-eCD3- | 18 | 24.3 | 8 |
| 16 | BM | male | 36 | new diagnosed | M5 | 121.84 | 20.6 | NPM1+ | CD117+CD38+CD123dim+CD13+CD33+MPO+HLA-DR+CD64+CD14+CD11e+CD11b+CD15+CD4(dim)+CD34-CD14-CD11e-CD11b-CD10-CD20-CD15- CD3-CD19-CD56-CD4-CD8-CD5-CD22-CD7-CD2-TdT-eCD3-D117-CD123-CD16- | 23.8 | 38.6 | 6 |
| 17 | BM | male | 14 | new diagnosed | M6 | 205.91 | 13 |  | CD34+CD117+CD33+CD64+CD13+CD11b+MPO+HLA-DR+CD38+CD15-CD14-CD123-CD11c-CD16-CD10-CD20-CD3-CD19-CD56-CD4-CD8-CD5-CD7-CD2-CD22-TdT-cCD3- | 36.4 | 90.8 | 45.8 |
| 18 | BM | female | 41 | new diagnosed | M5 | 60.58 | 29.7 |  | CD117+CD33+CD64+CD38+MPO+CD34+HLA-DR-CD15-CD14-CD123-CD11c-CD11b-CD13-CD16-CD10-CD20-CD3-CD19-CD56-CD4-CD8-CD5-CD7-CD2-CD22-TdT-cCD3-CD96- | 1 | 1 | 3 |
| 19 | BM | male | 13 | new diagnosed | M5 | 6.19 | 18 |  | CD117+CD15dim+CD13+CD33+MPO+HLA-DR+CD64+CD38+CD123dim+CD34+CD11b+CD14-CD11c-CD16-CD10-CD20-CD3-CD19-CD56-CD4-CD8-CD5-CD22-CD7-CD2-TdT-cCD3- | 93.6 | 97.8 | 56.9 |
| 20 | BM | male | 73 | new diagnosed | M5 | 2.92 | 80.5 |  | CD117+CD34+CD15dim+CD13+CD33+HLA-DR+CD64+CD38+CD56+MPO+TdT+CD11b+CD16-CD10-CD20-CD14-CD11c-CD123-CD4-CD8-CD3-CD19-CD2-CD5-CD7-CD22-cCD3- | 97.4 | 50.9 | 92.3 |
| 21 | BM | male | 18 | new diagnosed | M3 | 2.19 | 45 |  |  | 68.3 | 82.7 | 45.5 |
| 22 | BM | female | 75 | new diagnosed | M5 | 7.11 | 66 |  | CD117+CD15dimCD33+CD64+CD38+CD4+MPO+CD13+HLA-DR+CD34-CD16-CD10-CD20-CD123-CD11c-CD14-CD56-CD8-CD3-CD19-CD7-CD2-CD22-CD5-TdT- | 10.3 | 58.4 | 6.5 |
| 23 | BM | male | 17 | relapse | M2 |  | 10.1 | AML1-ETO+ | CD117+CD34+CD15dimCD33+HLA-DR+CD38+CD4dimCD56+MPO+CD13+CD64+CD11b-CD16-CD10-CD20-CD14-CD11c-CD3-CD19-CD8-CD5-CD22-CD2-CD7-TdT-cCD3- | 59.7 | 54 | 14.2 |
| 24 | BM | male | 51 | new diagnosed |  |  |  |  |  | 72.5 | 66.5 | 2 |
| 25 | BM | male | 62 | new diagnosed | M5 | 18.75 | 68.5 |  | CD117+CD34+CD13+CD33+HLA-DR+CD64+CD38+CD7+MPO+CD11b+CD15-CD16-CD10-CD20-CD14-CD11c-CD123-CD4-CD8-CD56-CD3-CD19-CD2-CD5-CD22-TdT-eCD3- | 61.7 | 95.6 | 74.4 |
| 26 | BM | male | 41 | new diagnosed |  |  |  |  |  | 24.9 | 88.3 | 87.4 |
| 27 | BM | male | 55 | new diagnosed | M2 | 33.46 | 81 |  |  | 0.5 | 62.9 | 3 |
| 28 | BM | male | 61 | new diagnosed |  |  |  |  |  | 83.3 | 83.7 | 58.5 |
| 29 | BM | female | 57 | new diagnosed | M2 | 2.12 | 47.5 | 46，XX；  CEBPA [mutation](javascript:void(0);) | CD34-CD117+CD33+CD13+HLA-DR+CD14-CD64-CD36-CD56-CD19-CD7+CD5- | 14.8 | 47.7 | 18 |
| 30 | BM | female | 56 | new diagnosed | M5 | 18.2 | 80 |  |  | 8.1 | 65.7 | 95.8 |
| 31 | BM | male | 18 | relapse | M5 | 6.5 | 69 |  |  | 50.1 | 34 | 85.1 |
| 32 | BM | female | 27 | new diagnosed |  |  |  |  |  | 1 | 1 | 3 |
| 33 | BM | male | 15 | new diagnosed | M1 | 30.41 | 86 | FLT3/KRAS/PTPN11/FAT1+ETO/MLL/CBFB- |  | 7.86 | 17.6 | 24.3 |
| 34 | BM | male | 33 | new diagnosed | M2 | 6.7 | 70 | 46，XY/45,XY,-10；AML/ETO- | CD33+CD13+HLA-DR+CD56+CD38+CD123+CD117+MPO+CD34+CD7+CD15+ | 48.2 | 27.5 | 36.8 |
| 35 | PB | male | 54 | new diagnosed | M5 | 3.09 | 22.2 |  | CD34+CD117+CD13+CD38+HLA-DR+CD11b+CD11c+TdT+CD15-CD16-CD10-CD20-CD64-CD14-CD4-CD8-CD56-CD3-CD19-CD7-CD22-CD2-CD5-CD123-CD33-cCd3-MPO- | 61.3 | 59.4 | 22.2 |
| 36 | PB | male | 33 | new diagnosed | M2 | 6.7 | 70 | AML/ETO- | CD33+CD13+HLA-DR+CD56+CD38+CD123+CD117+MPO+CD34+CD7+CD15+ | 33.1 | 30.1 | 40 |
| 37 | BM | female | 66 | new diagnosed | M4 | 43.82 | 22 |  |  | 65.5 | 83 | 44.2 |
| 38 | BM | male | 36 | relapse |  |  |  |  |  | 87.3 | 87.3 | 84 |
| 39 | BM | male | 15 | new diagnosed | M1 | 48.46 | 79 | FLT3/KRAS/PTPN11/FAT1+ETO/MLL/CBFB- |  | 63.7 | 68.7 | 55.7 |
| 40 | BM | male | 27 | new diagnosed | M5 | 140.9 | 36.2 |  | CD33+CD13+CD15dim+CD34+CD117+HLA-DR+CD7+MPO+CD64+CD56+CD15+CD16+CD11b-CD16-CD10-CD20-CD14-CD11e-CD4-CD8-CD3-CD19-CD2-CD5-TdT-eCD3-CD117-CD34-HLA-DR-CD20-CD11c-CD22-CD7- | 63.3 | 63.2 | 25.9 |
