# Supplementary figures and images for: CAR-T cells targeting CLL-1 as an approach to treat acute myeloid leukemia
Source: J Hematol Oncol. 2018 Jan 10;11:7. doi: 10.1186/s13045-017-0553-5 (PMC5761206; doi:10.1186/s13045-017-0553-5)

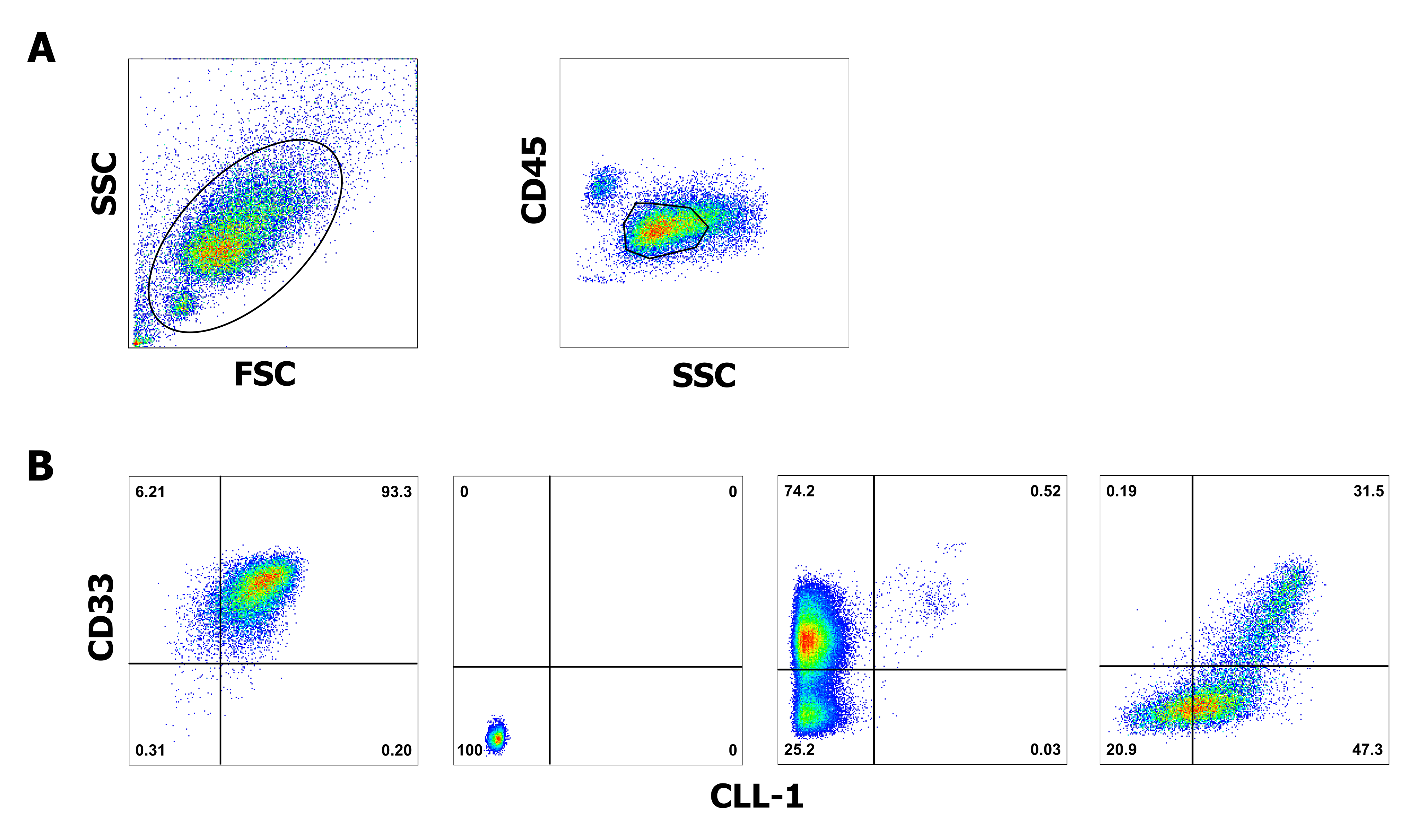

Supplement: Supplementary file 2 — Co-expression CLL-1 and CD33 in primary AML samples. (A) Initially, cells were gated based on forward and side scatter properties. Subsequently, AML blasts were selected based on low side scatter versus CD45dim expression. (B) CLL-1 and CD33 expression on four representative gated AML blast cell populations are depicted. Percentages in each quadrant are indicated. (TIFF 1776 kb) [file 13045_2017_553_MOESM2_ESM.tif]

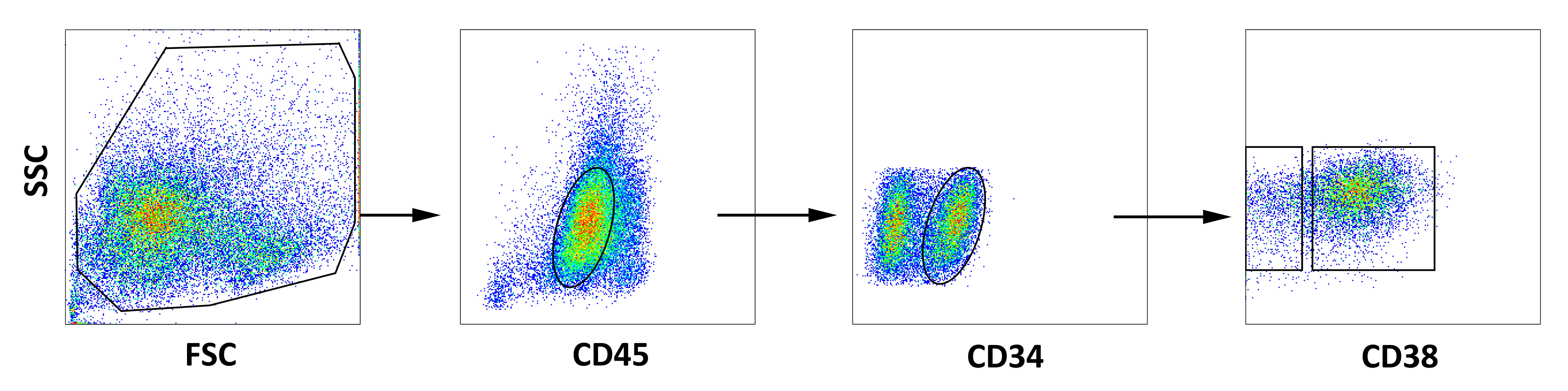

Supplement: Supplementary file 3 — The gating strategy of CD34+ AML blasts. Cells were initially gated based on forward and side scatter properties. Subsequently, AML blasts were selected based on low side scatter versus CD45dim expression. Then, CD34+ cells were gated. Finally, CD38+/CD38− cells were gated and used for CLL-1 expression analysis. (TIFF 1656 kb) [file 13045_2017_553_MOESM3_ESM.tif]

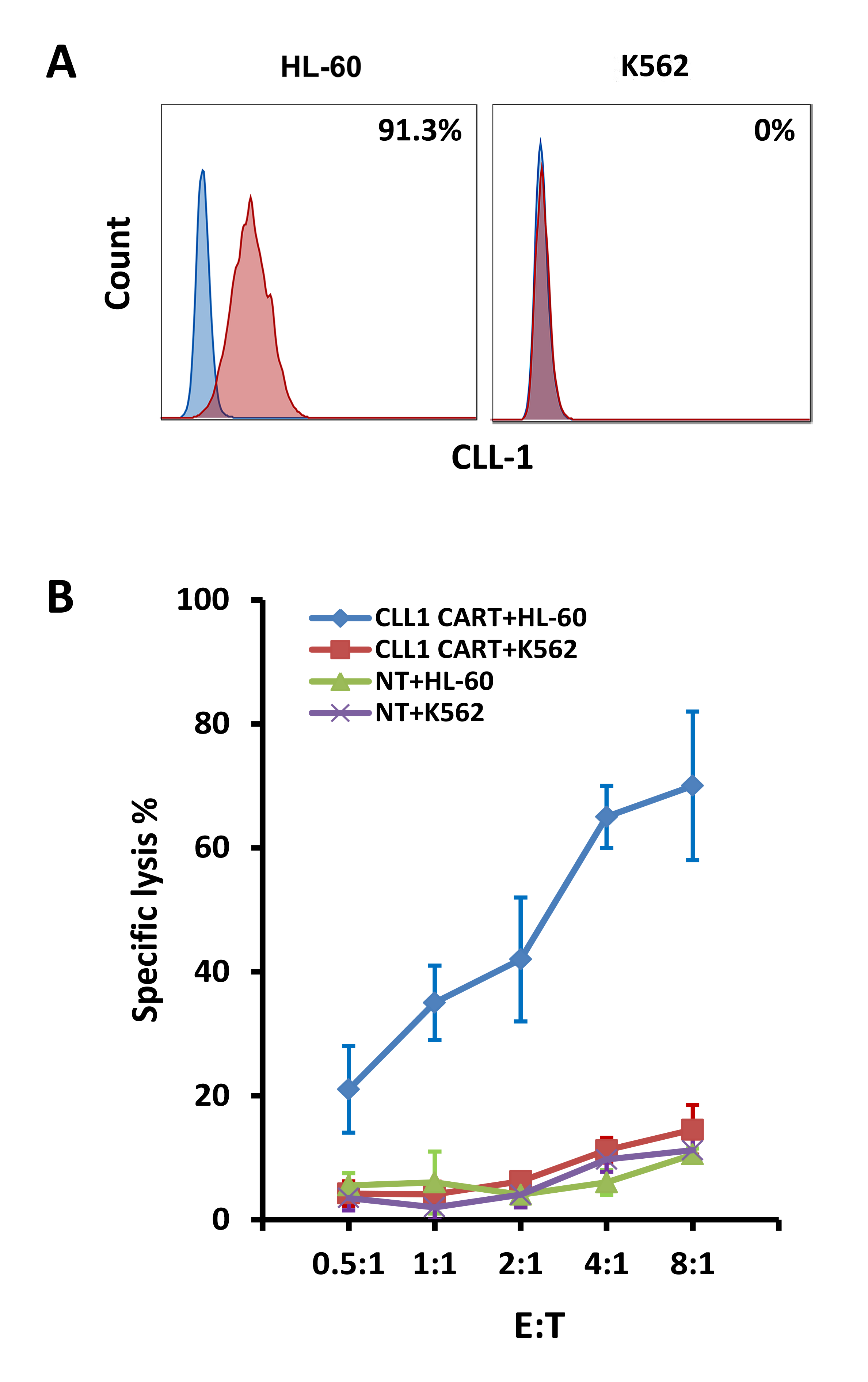

Supplement: Supplementary file 4 — CLL-1 CAR-T cells lyse CLL1-expressing AML cells. (A) Expression of CLL-1 on the cell lines HL-60 and K562. (B) CLL-1 CAR-T cells lysed CLL-1+ cell line HL-60. CLL-1− cell line K562 was used as negative control. NT cells were used to evaluate unspecific lysis. Data represent mean values of triplicate wells ± SD. (TIFF 481 kb) [file 13045_2017_553_MOESM4_ESM.tif]

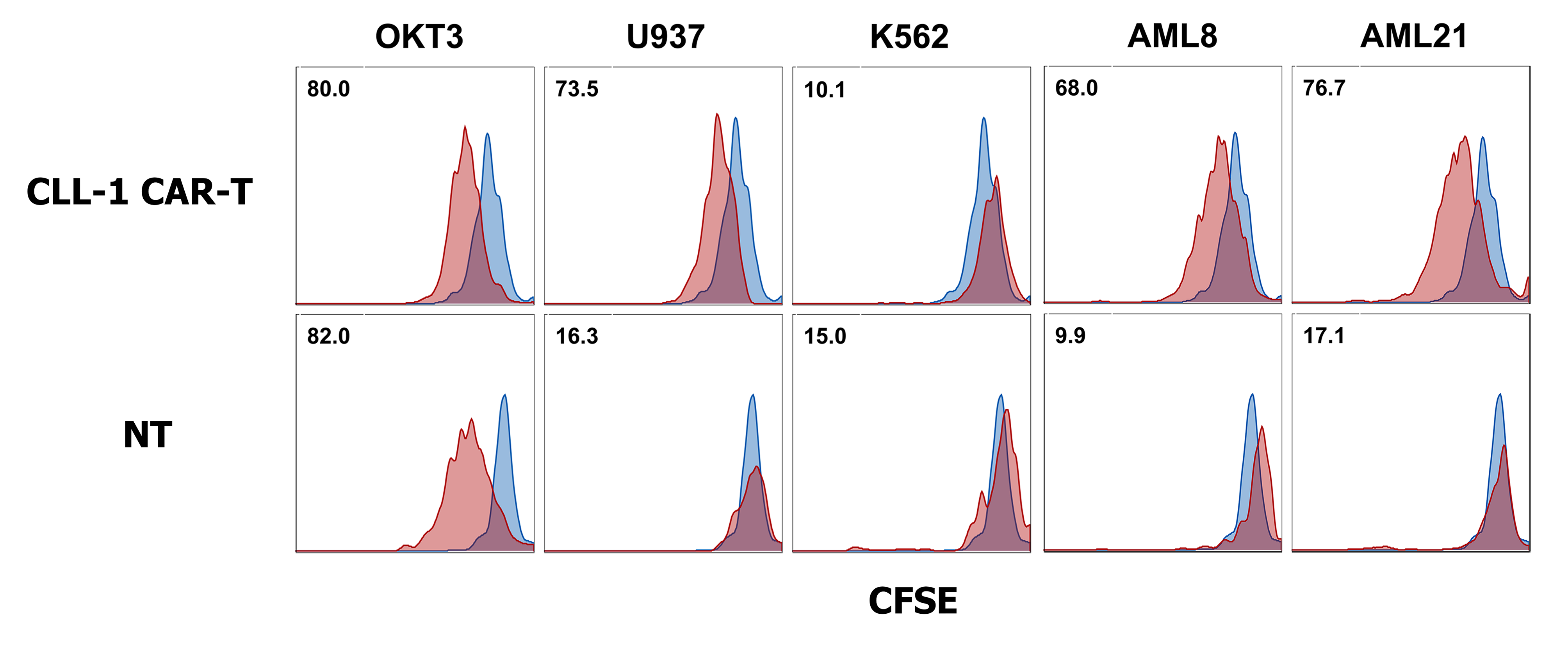

Supplement: Supplementary file 5 — Proliferation of CLL-1 CAR-T cells in response to CLL-1+ cells. Pair-matched CFSE-labeled CLL-1 CAR-T cells or NT cells were co-cultured with the indicated stimulator cell lines for 96 h at an E:T of 1:1. CFSE dilution was analyzed by flow cytometry. Unstimulated T cells (gray histograms) were used as baseline T cell proliferation controls. (TIFF 866 kb) [file 13045_2017_553_MOESM5_ESM.tif]

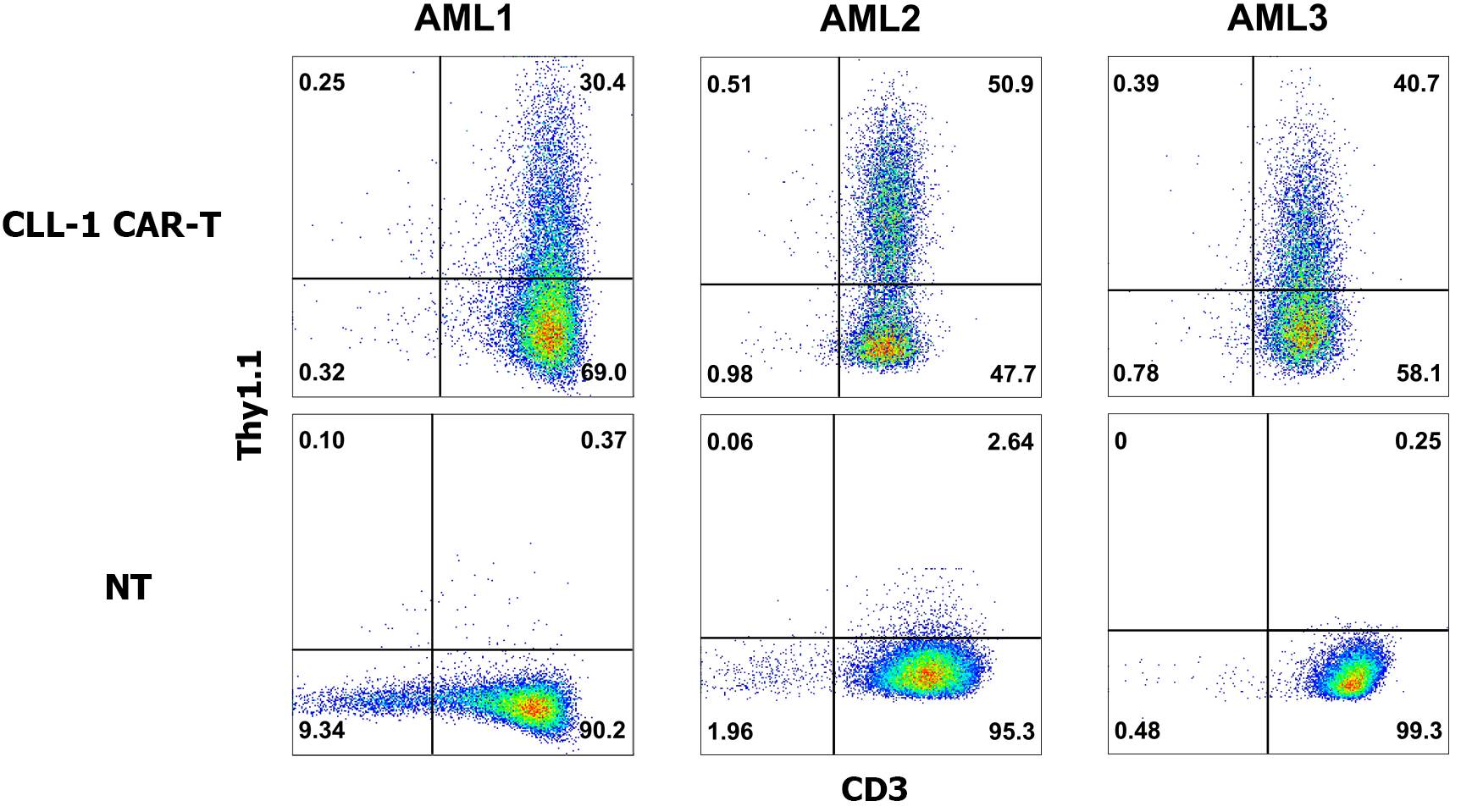

Supplement: Supplementary file 6 — CLL-1 CAR expression in T cells derived from AML patients. T cells from three AML patients were transduced with CLL-1 CAR. Shown are CLL-1 CAR-T and NT cells from the three AML patients 14 days post transduction. Percentages in each quadrant are indicated. (TIFF 557 kb) [file 13045_2017_553_MOESM6_ESM.tif]

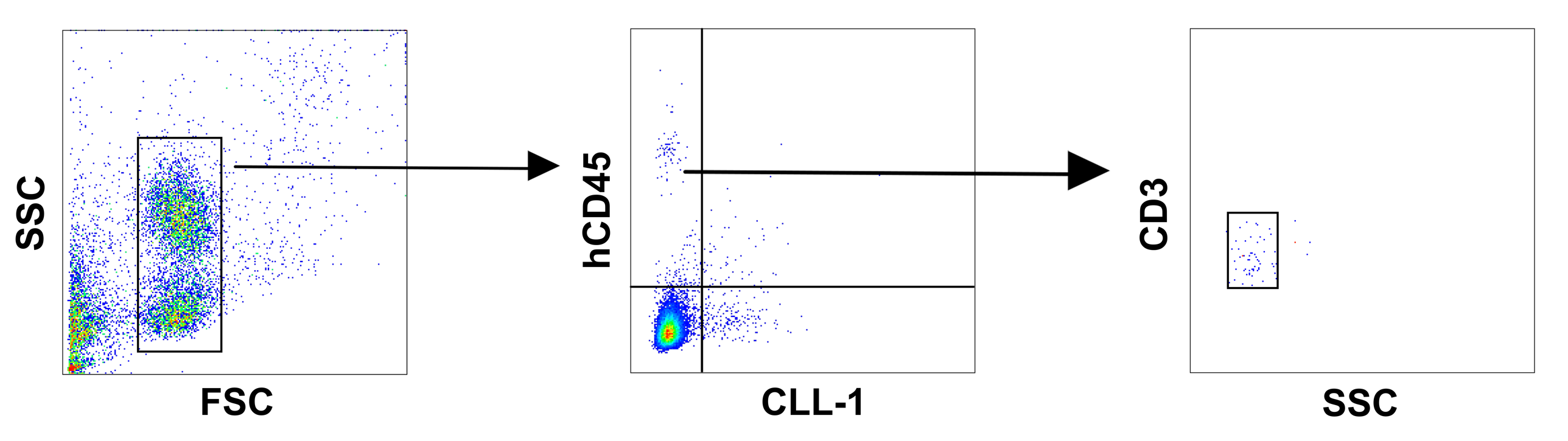

Supplement: Supplementary file 7 — Representative flow cytometric analysis of peripheral blood of CAR-T-treated mice. Eighteen days after leukemia transplant, hCD45+ CLL1− population in peripheral blood of CAR-T-treated mice was almost human T cells (hCD45+ CD3+). (TIFF 4614 kb) [file 13045_2017_553_MOESM7_ESM.tif]
